# Diagnostic efficacy of routine contrast-enhanced abdominal CT for the assessment of osteoporosis in the Turkish population

**DOI:** 10.3906/sag-1906-34

**Published:** 2020-02-13

**Authors:** Ayşegül CANSU, Dilara ATASOY, İlker EYÜBOĞLU, Murat KARKUCAK

**Affiliations:** 1 Department of Radiology, Faculty of Medicine, Karadeniz Technical University, Trabzon Turkey; 2 Department of Radiology, Sivas Numune Hospital, Sivas Turkey; 3 Department of Physical Medicine and Rehabilitation, Faculty of Medicine, Karadeniz Technical University, Trabzon Turkey

**Keywords:** Contrast-enhanced CT, bone mineral density, dual-energy X-ray absorptiometry, osteoporosis

## Abstract

**Background/aim:**

This study aimed to evaluate the diagnostic efficacy of vertebral Hounsfield unit (HU) values on routine contrast-enhanced abdominal computed tomography (CT) scans for the assessment of osteoporosis using dual-energy X-ray absorptiometry (DXA) T-scores as a reference standard.

**Materials and methods:**

A total of 111 consecutive patients who underwent contrast-enhanced abdominal CT examination for any indication and DXA within a 6-month period were retrospectively analyzed. The CT attenuation values of trabecular bone in HUs were measured in axial and sagittal planes from lumbar vertebrae 1–4 (L1–L4). The correlation between the DXA T-scores and HU values measured on the CT scans was evaluated by Pearson’s correlation test. Areas under the curves (AUCs) were calculated by receiver operating characteristic analysis for diagnostic proficiency, and threshold values were determined. Paired t-test and Bland–Altman plot test were used to evaluate the correlation between axial and sagittal HU values.

**Results:**

There was a strong correlation between the DXA T-scores and HU values of all the lumbar vertebrae (P < 0.001). The highest correlation was for the L3 vertebra; L3 was thus chosen for additional analyses. The mean axial and sagittal L3 attenuations were 133.7 HU and 131.9 HU, respectively. The axial measurements were not significantly different from the sagittal measurements, with a mean difference of 1.8 HU (P > 0.05). The L3 axial CT attenuation threshold for 90% sensitivity was 170 HU and that for 90% specificity was 102 HU for distinguishing osteoporosis from osteopenia and normal bone mineral density (BMD). To distinguish the low BMD group from the normal group, the L3 axial CT attenuation threshold for approximately 90% sensitivity was 102 HU and for 90% specificity was 165 HU.

**Conclusion:**

The HUs derived from routine contrast-enhanced abdominal CT scans can be used for the evaluation of osteoporosis, without additional radiation exposure and cost.

## 1. Introduction

Osteoporosis is a common systemic skeletal disorder characterized by reduced bone mineral density (BMD) and microarchitectural deterioration of the bone tissue, leading to an increase in bone fragility fractures [1]. Osteoporosis is one of the most important health problems owing to its high prevalence, mortality, and morbidity [2]. There are 200 million people worldwide with osteoporosis, and this prevalence is expected to rise by 310% and 240% in men and women, respectively, by 2050 [3,4]. Beyond the age of 50 years, half of all women and one quarter of men are known to suffer from an osteoporotic fracture in their lifetime, with most of the fractures occurring in patients not undergoing specific treatment for osteoporosis [5].

Currently, BMD measurement by dual-energy X-ray absorptiometry (DXA) is the gold standard method to evaluate osteoporosis [6]. However, more than 80% of the patients with osteoporotic fractures do not undergo BMD assessment or treatment to reduce the risk [7]. Moreover, this technique has some limitations such as false-negative results in patients with vertebral compression fractures, and limited use in people who have a spinal deformity or history of previous surgery [8]. Therefore, alternative methods are needed for osteoporosis screening.

Abdominal computed tomography (CT) performed for other indications offers helpful data about BMD. The BMD measurement from routine abdominal CT can be simply used without any additional patient radiation, time, and cost [9–13]. A systematic review has reported 37 studies evaluating the use of CT imaging for osteoporosis screening [14]. Most of these studies were performed with unenhanced CT and on axial vertebral planes [14]. However, only a few studies have reportedly investigated the utility of contrast-enhanced CT, which is more widely used than unenhanced CT for the assessment of osteoporosis [12,15–17]. The purpose of this study was to evaluate the diagnostic efficacy of vertebral Hounsfield unit (HU) values on routine contrast-enhanced abdominal CT scans for the assessment of osteoporosis using DXA T-scores as a reference standard. Additionally, we investigated the consistency between the measurements of sagittal and axial planes.

## 2. Materials and methods

### 2.1. Subjects

The patients who had undergone a DXA and contrast-enhanced abdominal CT examination between April 2014 and November 2017 were retrospectively analyzed in this study. All the examinations were performed at Karadeniz Technical University’s Faculty of Medicine. The contrast-enhanced abdominal CT was performed for various routine clinical indications, most commonly oncological ones. Moreover, the patients with a time lapse of >6 months between the CT and DXA were not included. The study was approved by the local ethical committee, and a need for informed consent was waived since retrospective data were used.

### 2.2. Dual-energy X-ray absorptiometry 

DXA of the lumber spine was used as a standard technique. All DXA scans were performed on the spine [lumbar vertebrae 1–4 (L1–L4)] using standard techniques on Lunar Prodigy densitometers (GE Healthcare, Milwaukee, WI, USA). The patients were classified as osteoporotic (T score ≤ −2.5), osteopenic (−2.5 < T score < −1.0), and normal (T score ≥ 1.0), based on the DXA T-scores.

### 2.3. Computed tomography

**Figure 1:**
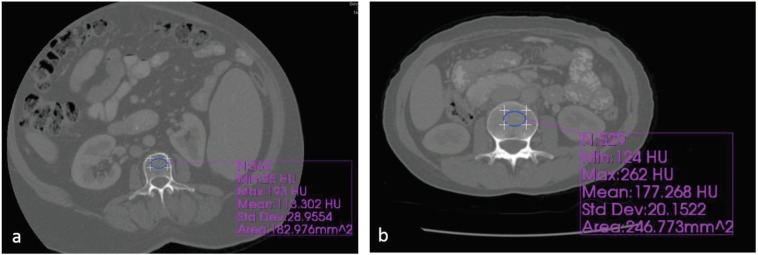
Contrast-enhanced abdominal CT scans showed density measurement from L3 vertebra on the axial plane. The patient on the left (a) is in the osteoporotic group, below the threshold (balanced threshold = 119), while the patient on the right (b) is in the normal group, above the threshold (balanced threshold = 133). According to the DXA T-scores, the patient on the left (a) is osteoporotic, and the patient on the right (b) has a normal T score.

**Figure 2:**
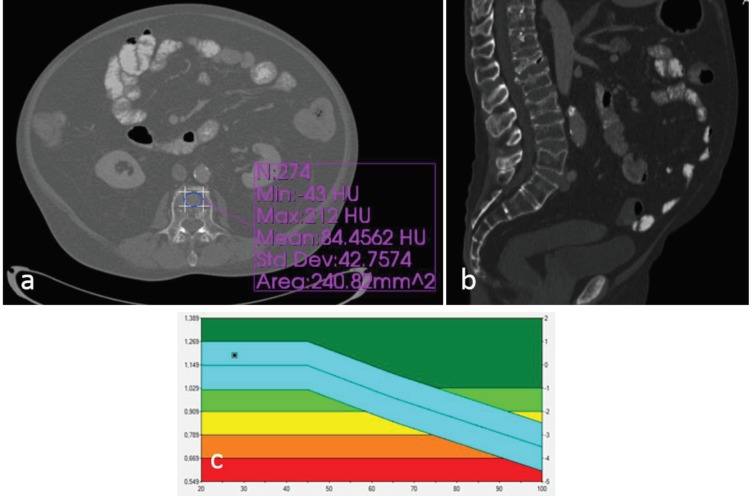
A patient with osteoporotic vertebrae fractures. a) The abdominal CT density measurement from L3 vertebra on axial plane showed osteoporotic HU values (84 HU). b) Sagittal plane CT image showed osteoporotic collapse fractures at lumbar vertebrae. c) However, the patient had normal T-score, according to the DXA T-scores.

All CT examinations were performed using a 160-slice dual-energy CT scanner (Toshiba Aquilion, Toshiba Medical Systems, Tochigi, Japan). Axial images were obtained with thin collimation at 120 kVp during portal venous phase (start delay, 70 s) and reconstructed at 2-mm thickness. Density values were measured on the picture archiving and communication system workstation by the agreement of two radiologists who were blinded to the DXA results. CT attenuation values (in HU) were determined on axial planes of bone from L1 to L4. An oval-shaped region of interest (ROI) was used on both bone and soft tissue windows. The smallest ROI size was 170 mm2 and the largest ROI size was 250 mm2. When the ROIs were placed, the midline of the vertebrae was selected, avoiding the posterior venous plexus zones, focal heterogeneous fields or lesions, and imaging-related artifacts (Figure 1). This process was subsequently performed on the sagittal reconstructions without knowledge of HU values in the axial planes. No measurements were made from compression fractures. Existence of moderate–severe compression fractures was evaluated using the sagittal CT images (Figure 2).

### 2.4. Statistical analysis

The correlation between the lumbar vertebrae DXA T-scores and the HU values measured in the CT scan was evaluated by Pearson’s correlation test. Areas under the curves (AUCs) were calculated by receiver operating characteristic (ROC) analysis for diagnostic proficiency. We calculated the sensitivity, specificity, and positive and negative predictive values of abdominal CT imaging. Threshold values were determined in two different comparisons. The first was calculated to separate the nonosteoporotic group (normal and osteopenic) from the osteoporotic group, and the second one was calculated to separate the normal from the low BMD (osteoporotic and osteopenic) group. For all the comparisons, threshold values were determined for high sensitivity, high specificity, and balanced sensitivity–specificity. We used a paired t-test and Bland–Altman plot test to evaluate the correlation between axial and sagittal HU values. P < 0.05 was considered statistically significant. Statistical analysis was performed using SPSS 13 (SPSS Inc., Chicago, IL, USA).

## 3. Results

A total of 111 patients had undergone DXA and contrast-enhanced abdominal CT within the 6-month period. Of all the patients, 73% were female (n = 81) and 27% were male (n = 30). The mean age was 57.6 years (16–87 years, standard deviation (SD) = 14.8 years). The mean time between the abdominal CT and DXA examination was 53.7 days (0–180 days, SD = 50.9 days). Contrast-enhanced abdominal CT was performed most commonly for oncologic follow-ups, with the second most common reason being a traumatic work-up.

According to the reference L1–L4 mean T-scores, 62 (55.9%) of the 111 patients were normal, 31 (27.9%), osteopenic, and 18 (16.2%), osteoporotic. Four out of the 111 patients (three female patients and one male) had at least one moderate or severe fracture in the spine, with three of them having an osteoporotic T score; however, one of them had a normal T score (Figure 2).

**Figure 3:**
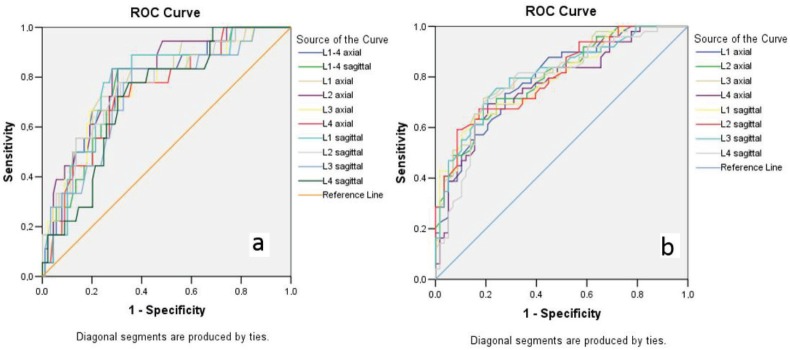
ROC curves of both axial and sagittal HU values for L1–L4 vertebrae to predict osteoporosis (a) and low BMD (b) based on the DXA T-scores.

By using both axial and sagittal attenuations for all the four single-level L1–L4 and the average of them, ROC curves were constructed to predict osteoporosis and low BMD, according to the DXA T-scores reference standard (Figures 3a and 3b). There was a strong correlation between the DXA T-scores and the mean HU values of all the lumbar vertebrae (P < 0.001). The highest correlation was evident for the L3 vertebra (sagittal plane r = 0.619, axial plane r = 0.615) and thus L3 was chosen for additional analyses. 

**Figure 4:**
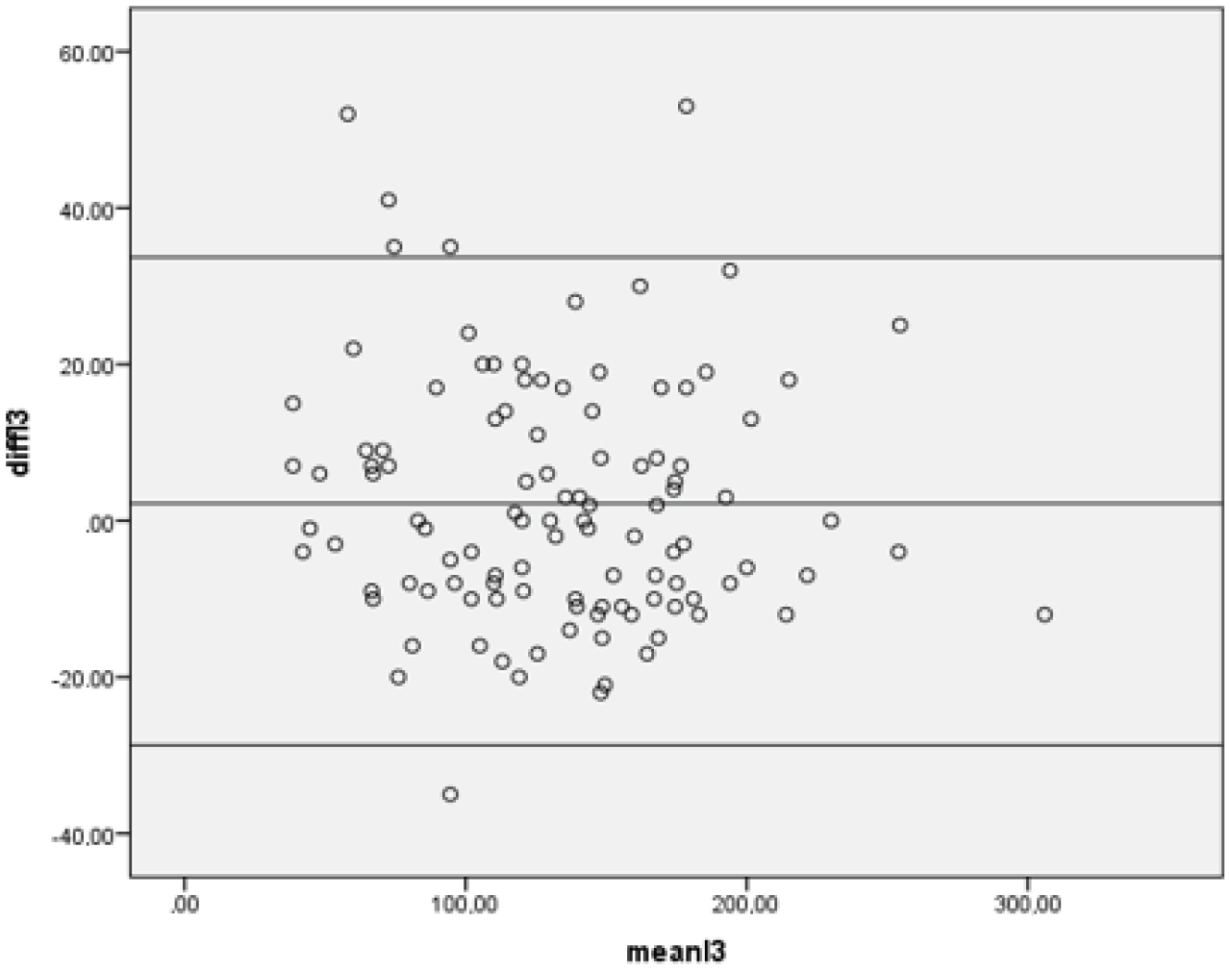
Bland–Altman plot of L3 axial and L3 sagittal HU values revealed good correlation between the two methods without any proportional bias.

The mean axial and sagittal L3 attenuations were 133.7 HU and 131.9 HU, respectively. The axial measurements were not significantly different from the sagittal measurements, with a mean difference of 1.8 HU (P > 0.05). A Bland–Altman plot test did not reveal any significant proportional bias of these attenuation differences on the average attenuation (Figure 4).

Threshold values for distinguishing the osteoporotic from nonosteoporotic (normal–osteopenic) groups and the normal from low BMD (osteoporotic and osteopenic) groups were calculated. The threshold of the L3 vertebra and mean value of L1–L4 vertebrae were separately calculated for high sensitivity (approximately 90%), high specificity (approximately 90%), and average sensitivity–specificity (approximately 70%) (Tables 1 and 2).

**Table 1:** The threshold values for comparison of osteoporotic–nonosteoporotic groups.

	High sensitivity (about 90%)			High specificity (about 90% )			Balanced sensitivity and specificity		
Vertebraelevel	Threshold (HU)	Sensitivity(%)	Specificity (%)	Threshold (HU)	Sensitivity (%)	Specificity(%)	Threshold (HU)	Sensitivity(%)	Specificity(%)
L3 axial	≤170	88.9	25.3	≤102	66.7	89.1	≤119	72.2	65.9
L3 sagittal	172	88.9	21.1	93	44.4	80	122	77.8	63
L1–4 axial	159	88.9	34.4	90	50	86.7	121	77.8	68.9
L1–4 sagittal	171	88.9	28.1	94	44.4	80.9	121	70.8	77.8

**Table 2:** The threshold values for comparison of normal-low BMD (osteopenic–osteoporotic).

	High sensitivity (about 90%)			High specificity (about 90% )			Balanced sensitivity and specificity		
Vertebraelevel	Threshold (HU)	Sensitivity(%)	Specificity (%)	Threshold (HU)	Sensitivity (%)	Specificity(%)	Threshold (HU)	Sensitivity(%)	Specificity(%)
L3 axial	≥102	90	51	≥165	38	89.8	≥133	70	75
L3 sagittal	102	89.8	49	170	33.9	89.8	133	69.5	75.5
L1–4 axial	106	88	51	167	35.6	89.8	140	69.5	79.6
L1–4 sagittal	111	89.7	55.1	172	36	87.8	141	70.7	73.5

An L3 axial CT attenuation threshold for 90% sensitivity was 170 HU and for 90% specificity was 102 HU, with a threshold of 119 HU resulting in balanced sensitivity and specificity (about 65%–70% for distinguishing osteoporosis from osteopenia and normal BMD (Table 1).

To distinguish the low BMD group from the normal group, an L3 axial CT attenuation threshold for 90% sensitivity was 102 HU and for 90% specificity was 165 HU (Table 2). A threshold of 133 HU resulted in a balanced sensitivity and specificity (about 70%–75%) (Table 2).

## 4. Discussion

In the present study, we found a strong correlation between the DXA T-scores and HU values obtained from routine contrast-enhanced abdominal CT of all the lumbar vertebrae. The highest correlation was seen with the L3 vertebra. Furthermore, we compared HU values of axial and sagittal planes and did not find a significant difference. Thus, our study findings showed that opportunistic vertebral attenuation values for osteoporosis screening and compression fracture assessment can be performed simply on the same view on abdominal CT.

Many studies have evaluated the utility of unenhanced CT and quantitative CT (QCT) for osteoporosis screening [14]. However, few studies have compared vertebral attenuation values on contrast-enhanced CT with the BMD measurements on DXA [12,15–17]. The QCT requires advanced technical software and a lot of time for obtaining BMD measurements [18]. Thus, it is not a practical opportunistic method for the assessment of osteoporosis. Unenhanced CT images provide reliable density values [14], but in clinical practice, a high number of CT examinations are performed using contrast agents for oncologic follow-ups. Owing to these limitations, the HU values should be obtained from the contrast-enhanced CT scans for opportunistic osteoporosis screening. In our study, we compared the vertebral attenuation values on contrast-enhanced abdominal CT images with the DXA T-scores. There was a strong correlation between the DXA scores and HU values of all the lumbar vertebrae, as exhibited in previous studies [12,15,17]. Any vertebral level from L1 to L4 can be used for the evaluation of osteoporosis without a statistically significant difference [12,15]. However, we found the highest correlation for the L3 vertebra, as opposed to the findings of other studies [12,15]. Pickhardt et al. highlighted the L1 measurements, and Kara et al. found the highest correlation between L4 and L5 [12,15,19]. Thus, we chose the L3 vertebra measurements for additional analyses.

The optimal thresholds of vertebral HU values were not identical among the studies [14]. In our study, the L3 vertebra attenuation values had the highest correlation for the DXA T-scores. The mean axial and sagittal L3 attenuation values were 133.7 HU and 131.9 HU, respectively. We found that an L3 axial CT attenuation threshold of 170 HU was about 90% sensitive, and a threshold of 102 HU was about 90% specific for distinguishing osteoporosis from osteopenia and normal BMD. For distinguishing the low BMD group from the normal group, our results indicated that thresholds of 102 HU and 165 HU were approximately 90% sensitive and 90% specific, respectively. These values slightly differed from the previous contrast-enhanced studies, where the L1 vertebra was chosen for analyses of the threshold values [12,15,17]. Alacreu et al. [17] reported an L1 attenuation threshold of 160 HU to be 90% sensitive and a threshold of 73 HU to be 90% specific for distinguishing osteoporosis from nonosteoporosis. Pickhardt et al. reported two studies at different times and with different colleagues [12,15]. In the first study, 55% of the CT scans were obtained after intravenous contrast injection [12]. The AUCs were statistically similar for contrast-enhanced and unenhanced CT scans, and they considered that the accuracy of BMD measurements was unaffected by the presence or absence of intravenous contrast [12]. Further, they established an L1 CT attenuation threshold of 160 HU as 90% sensitive and a threshold of 110 HU as >90% specific for distinguishing osteoporosis from nonosteoporosis. In the later study, patients who had both unenhanced and contrast-enhanced abdominal CT scans and had undergone DXA were included for the assessment of osteoporosis [15]. The mean difference between unenhanced and contrast-enhanced CT examinations was found to be 11 HU. The L1 threshold of contrast-enhanced CT scans for 90% sensitivity was 144 HU and for 90% specificity was 102 HU for diagnosing osteoporosis [15]. The AUCs for both unenhanced and contrast-enhanced abdominal CT series were similar at 0.81 and 0.83, respectively. The results of these studies and our study show that contrast-enhanced CT scans have the potential to reliably diagnose osteoporosis.

In this study, we also revealed a close agreement between the axial and sagittal CT attenuation values. To the best of our knowledge, only Lee et al. compared the axial and sagittal CT attenuation values on routine abdominal CT [20]. Similar to our findings, they found that both these measurements matched well. This result shows that the evaluation of vertebral fracture and osteoporosis screening can be combined using the sagittal reconstruction only, and this saves time in busy clinical practice.

The present study has some limitations. First, this was a retrospective single-center study. Furthermore, the patient population was heterogeneous in terms of indication for CT. The thresholds were study-dependent, thus dictating a need for larger cohorts to be more reliable. Second, patients who underwent contrast-enhanced CT were included in this study, and the impact of intravenous contrast material on CT attenuation value was not assessed. Finally, interobserver variability was not also evaluated.

In conclusion, the HUs derived from routine contrast-enhanced abdominal CT scans can be used for the evaluation of osteoporosis, without additional radiation exposure and cost. A close agreement between the axial and sagittal plane measurements showed that BMD measurements and osteoporotic vertebral fracture assessment can be combined simply on the sagittal view alone. Further investigation with larger sample size is required to obtain specific CT attenuation thresholds. 

## Informed consent

The present study was approved by the local ethical committee, and informed consent was waived since retrospective data were used (Number: 2018/111).

## Financial disclosure

The authors declare that this study has received no financial support.
